# Construction of an Interspecific Genetic Map Based on InDel and SSR for Mapping the QTLs Affecting the Initiation of Flower Primordia in Pepper (*Capsicum* spp.)

**DOI:** 10.1371/journal.pone.0119389

**Published:** 2015-03-17

**Authors:** Shu Tan, Jiao-Wen Cheng, Li Zhang, Cheng Qin, Ding-Guo Nong, Wei-Peng Li, Xin Tang, Zhi-Ming Wu, Kai-Lin Hu

**Affiliations:** 1 College of Horticulture, South China Agricultural University, Guangzhou, China; 2 Pepper Institute, Zunyi Academy of Agricultural Sciences, Zunyi, Guizhou, China; 3 Maize Research Institute of Sichuan Agricultural University / Key Laboratory of Biology and Genetic Improvement of Maize in Southwest Region, Ministry of Agriculture, Chengdu, China; 4 College of Agriculture, Guangxi University, Nanning, China; 5 College of Horticulture and Landscape Architecture, Zhongkai University of Agriculture and Engineering, Guangzhou, China; The University of Western Australia, AUSTRALIA

## Abstract

Re-sequencing permits the mining of genome-wide variations on a large scale and provides excellent resources for the research community. To accelerate the development and application of molecular markers and identify the QTLs affecting the flowering time-related trait in pepper, a total of 1,038 pairs of InDel and 674 SSR primers from different sources were used for genetic mapping using the F_2_ population (n = 154) derived from a cross between BA3 (*C*. *annuum*) and YNXML (*C*. *frutescens*). Of these, a total of 224 simple PCR-based markers, including 129 InDels and 95 SSRs, were validated and integrated into a map, which was designated as the BY map. The BY map consisted of 13 linkage groups (LGs) and spanned a total genetic distance of 1,249.77 cM with an average marker distance of 5.60 cM. Comparative analysis of the genetic and physical map based on the anchored markers showed that the BY map covered nearly the whole pepper genome. Based on the BY map, one major and five minor QTLs affecting the number of leaves on the primary axis (Nle) were detected on chromosomes P2, P7, P10 and P11 in 2012. The major QTL on P2 was confirmed based on another subset of the same F_2_ population (n = 147) in 2014 with selective genotyping of markers from the BY map. With the accomplishment of pepper whole genome sequencing and annotations (release 2.0), 153 candidate genes were predicted to embed in the *Nle2*.*2* region, of which 12 important flowering related genes were obtained. The InDel/SSR-based interspecific genetic map, QTLs and candidate genes obtained by the present study will be useful for the downstream isolation of flowering time-related gene and other genetic applications for pepper.

## Introduction

*Capsicum* is a member of the Solanaceae family and consists of the following five most important cultivated species, *C*. *annuum*, *C*. *chinense* Jacq., *C*. *baccatum*, *C*. *pubescens* Ruiz & Pavon and *C*. *frutescens* [1]. Of which *C*. *annuum* is most widely cultivated for use as food, spice, ornaments, and medicine around the world. *C*. *frutescens* and *C*. *chinense* exhibit a relatively closer interspecific relationship with *C*. *annuum* and provide critical resources for the genetic improvement of pepper production [2,3]. Even though interspecific crosses suffer from low fertility and high segregation distortion, they benefit from higher level of polymorphism [4] and provide opportunities to introduce economically valuable traits into the cultivated species [5].

During the last few decades, genetic maps have become the basic tool necessarily for genetics and breeding such as genome assembly, QTL analysis, gene tagging and marker-assisted selection (MAS). Numerous genetic maps including integrated maps have been constructed for pepper [6,7] using either intraspecific [8–12] or interspecific populations [13–20]. In these studies, different marker systems such as Restriction Fragment Length Polymorphism (RFLP), Random Amplified Polymorphic DNA (RAPD), Amplified Fragment Length Polymorphism (AFLP), Simple Sequence Repeat (SSR) and Single Nucleotide Polymorphism (SNP) had been used. However, the number of simple PCR-based molecular markers for pepper remains to be increased [8,17]. Insertion/deletion (InDel) polymorphism, which is known as a user-friendly marker type, has high variability and co-dominant inheritance and is relatively abundant and uniformly distributed throughout the genome [21,22]. With the decreasing cost of next generation sequencing, InDels can be developed on a large scale through re-sequencing and are becoming a popular choice for plant and animal systems [21–25]. Unfortunately, InDel discovery efforts have lagged significantly behind SSR discovery efforts and relatively few InDels have been identified in pepper. In addition, to our knowledge, InDel markers have seldom been used for genetic mapping in spite of more intensive sequencing of pepper in recent years [26–29]. Fairly recently, we constructed an initial InDel-based genetic map (BB-InDel map) using an intraspecific population [30].

In flowering plants, the initiation of flower primordia indicates the start of the transition from the vegetative phase to the reproductive phase that will definitively reflect the flowering time, which is one of the most important economic traits in conventional pepper breeding [31]. Pepper is a member of the Solanaceae family and has a sympodial shoot structure [32]. The formation of flower primordia is controlled by the shoot apical meristem (SAM), which terminates in an inflorescence meristem (IM) that subsequently develops into a solitary flower along with the reproductive transition [33]. Up to now, several genes controlling the transition to flowering and shoot architecture were reported in pepper through EMS mutagenesis [33–37]. Of these, *Ca-ANANTHA* (*Ca-AN*), *CaBLAND* (*CaBL*), *CaJOINTLESS* (*CaJ*) and *Capsicum annuum S* (*CaS*) were all found to promote the early flower formation in pepper while *FASCICULATE* (*FA*) stimulated late flowering. In addition, both genes *CaHAM* and *CaBL* participated in the controlling of axillary meristem formation [38]. The relationships between these genes were also partly investigated. For example, *CaJ* showed epistasis over *FA* [36] and *CaBL* functions independently of *FA* in regulating sympodial growth, but is epistatic to *FA* in controlling axillary meristem formation [35]. Recent results also indicated that *CaS* is epistatic over other genes controlling the transition to flowering with respect to flower formation [33]. Even so, the molecular regulatory mechanism of pepper flowering primordia initiation is poorly understood. More importantly, the cause of wide natural variation in flowering time is still cryptic for pepper. In fact, pepper exhibits widespread natural variation in flowering time and the number of leaves on the primary axis (Nle) ranges from 1 to more than 20 in different species [39]. Classical quantitative genetic analysis and QTL mapping showed that Nle was commonly controlled by a few major genes with some minor factors, as well as the environment [12,31,39–44]. Additionally, so far QTLs affecting Nle had been identified on all pepper chromosomes with exception of P9 and P10 using different populations [31,40,44–46]. However, most of these studies were based on intraspecific populations.

In this study, a genetic linkage map was first constructed based on InDel and SSR markers using the F_2_ population derived from an interspecific cross between BA3 (*C*. *annuum*) and YNXML (*C*. *frutescens*). Subsequently, QTL analysis was performed to identify the genomic region associated with the flowering time-related trait (namely Nle) by using the two subsets of the same F_2_ population in 2012 and 2014, respectively. Finally, the candidate genes embed in the QTL region were discussed. The genetic map, QTLs and candidate genes therein this study will provide useful information for molecular assisted selection (MAS) breeding, and lay the foundation for the isolation of genes underlying the variation in flowering time in pepper.

## Materials and Methods

### Plant materials and trait evaluation

An F_2_ genetic mapping population was derived from the cross between the inbred lines BA3 (*C*. *annuum*) and YNXML (*C*. *frutescens*), both of which were re-sequenced [1]. BA3 is a cytoplasmic male sterility (CMS) line with Nle ranged from 8 to 12. YNXML, which is a pungent type with small size and erect fruit, was collected from Yunnan Province, China, and its Nle is approximately two times as BA3, which led directly to a flowering time that occurred 10~15 days later than BA3. The F_2_ population was divided into two sub-groups consisting of 154 and 147 individuals, respectively. Approved by the Office for Teaching & Research Bases, the two subsets, together with the parental lines and their hybrid population, were grown successively at the Zengcheng Experimental Station, South China Agricultural University (SCAU), Guangzhou, China (23° 083 N, 113mental) in 2012 and 2014, respectively. Genomic DNA was extracted from young leaves using the modified CTAB method [47]. The Nle were numbered successively from the node of the cotyledon to the first flower node on the main stem, as recommended by the IPGRI (The International Plant Genetic Resources Institute), for each individual plant after the formation of the first branch.

### Sources and genotyping of InDel and SSR

Information on the InDel and SSR primers with different sources [17,48–50] that were used in the present study are summarized in. A total of 1000 pairs of InDel primers recently developed from re-sequencing [30] were selected and used to screen the parental lines (BA3 and YNXML) for polymorphism. An additional 38 InDels, which have been predicted between BA3 and YNXML using the same bioinformatics analysis pipeline, were selected to increase the marker density of P2. In addition, a total of 420 EST-SSRs from a public database (http://compbio.dfci.harvard.edu/tgi/plant.html) were previously identified by our group [48], and also used for polymorphism screening in the present study. 119 primer pairs of genomic SSR markers and 135 primer pairs of EST-SSR markers that were previously reported (Table 1) were also used. A PCR mixture contained 10 ng genomic DNA, 200 μM of each dNTP, 2 μM of each primer, 1 × reaction buffer, 37.5 μM of Mg^2+^, and 0.5 unit of Taq polymerase (Dsbio) in a final volume of 20 μL. The reaction was performed as follows: an initial cycle of 5 min at 94°C; 34 cycles of 45 s at 94°C, 45 s at 58°, and 1 min at 72°m, and a final 10 min at 72°m.

**Table 1:** Polymorphism screening of primers from different sources.

Marker type	Sources	Prefixed	No. of primers	Successful amplification		Polymorphic		Sources
				N	Percentage (%)	N	Percentage (%)	
InDel	Re-sequencing	CIDH_	1,000	976	97.60	129	12.90	[30]
InDel	Re-sequencing	CIDHjw_	38	35	92.11	11	28.95	In present study
SSR	EST	PSE_	420	403	95.95	64	15.24	[48]
SSR	EST	EPMS_	135	130	96.30	22	16.30	[50]
SSR	Genome	GPMS_, Hpms_, AF_	119	113	94.96	16	13.45	[17,49]
Total	-	-	1,712	1657	96.79	242	14.14	-

### Genetic map construction and comparison with the physical map

A genetic map was constructed using JoinMap 4.0 software with a population type code, F_2_ [51]. Most of the InDel markers were grouped according to the physical mapping information [30]. The remaining InDel markers from P0 were assigned to the known groups using the *Strongest Cross Link* (SCL) information. Both the SCL information and BLAST tool [52] were used to map the SSR markers onto the pseudo chromosomes (groups) of the Zunla-1 reference genome (http://peppersequence.genomics.cn). Recombination values were converted to genetic distances using the Kosambi mapping function [53] and a comparative map was drawn using Mapchart 2.2 [54]. The segregation ratios of markers in the population were examined by Chi-square analysis. Markers with segregation ratios that differed from expected 1:2:1 or 3:1 at *P* <0.05 were classified as segregation distortion markers. Similar to the definition in previous study [55], a region with five or more adjacent skewed segregation markers was defined as a segregation distortion region (SDR) in present study.

### QTL analysis of Nle

Both of the Inclusive Composite Interval Mapping (ICIM) [56] and Composite Interval Mapping (CIM) was initially applied to detect QTLs (LOD > 2.5) for Nle with the sub-population (n = 154) of 2012 using the QTL IciMapping 4.0 and Windows QTL Cartographer 2.5 [57], respectively. Further, a set of 111 markers from the above map, which were uniformly distributed on the whole physical map but with preference to the chromosome P2, was selected to genotype another sub-population (n = 147) of 2014 and subsequently independent QTL analysis was performed as above to evaluate the primary QTL results of 2012. On the other hand, Single Marker Analysis (SMA) was carried out on a dataset that normalized from the phenotypic data of two years (2012 and 2014) with the selected positive markers from the 2012 analyses as well.

## Results and Discussion

### Polymorphism screening

In order to construct an interspecific genetic map based purely on user-friendly PCR markers, InDel and SSR primers from different sources (Table 1) were used for polymorphism screening between BA3 (*C*. *annuum*) and YNXML (C. *frutescens*). A total of 1,000 pairs of InDel primers that were developed previously by our group [30] and an additional 38 predictably polymorphic InDels between BA3 and YNXML were selected and then subsequently used to screen for polymorphism between the parental lines (BA3 and YNXML). Finally, 140 out of 1,038 pairs of InDel primers were validated and used for further genetic mapping (Table 1 and S1 Table.). On the other hand, 64 polymorphic SSR loci (S2 Table.) between BA3 and YNXML were identified from the 420 pairs of EST-SSR primers that developed by our group [48]. None of these EST-SSR markers have been used for genetic mapping and their rate of polymorphism (15.24%) is similar to previous reports (16.30%), and slightly higher than that of genome-derived SSRs (13.45%, Table 1). In total, 1712 pairs of PCR-based primers with different sources were analyzed and 242 pairs of polymorphic primers (14.14%) were validated and then subsequently applied to the genotyping of the F_2_ individuals (Table 1).

### Genetic map construction

**Fig 1:**
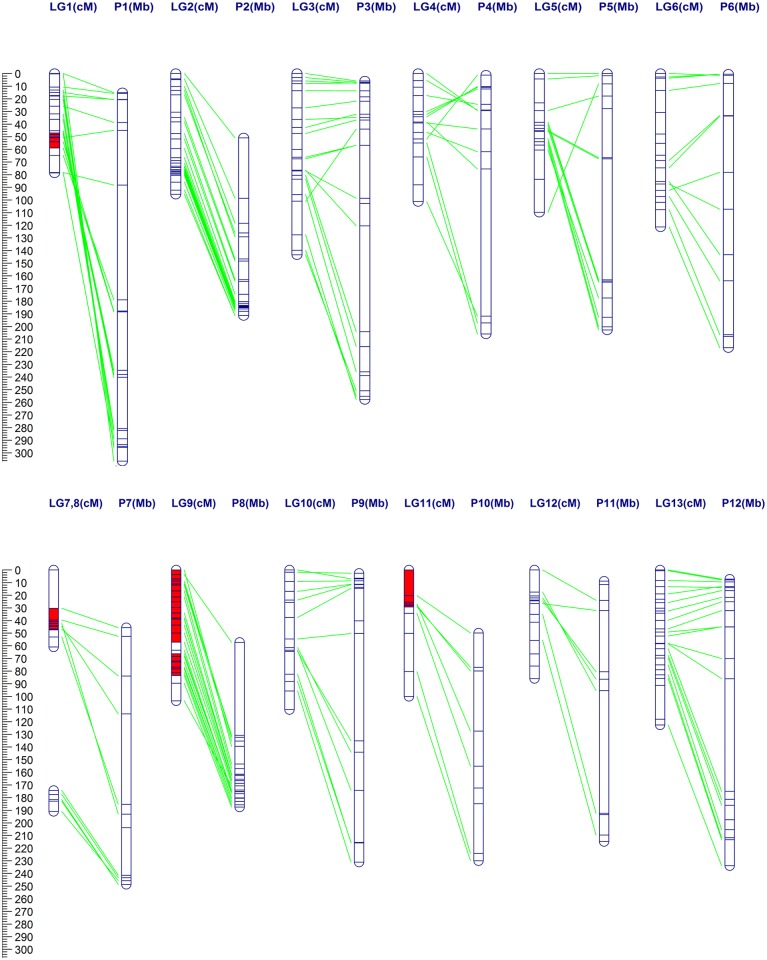
An interspecific genetic map of pepper based on 224 InDel and SSR markers, and the comparison with its physical map. A total of 13 LGs (LG1 ~ LG13) were assigned to the corresponding chromosomes (P1 ~ P12) based on anchored markers. P7 was divided into LG7 and LG8 due to insufficient linkage. Green lines indicate the synteny between the genetic and physical maps. Five SDRs on LG1 (= P1), LG7 (= P7), LG9 (= P8) and LG11 (= P10) are filled with a red color.

The F_2_ progenies were genotyped with the 242 markers, including the 140 InDels and 102 SSRs (S1 and S2 Tables.). Of these, 4 SSR markers with inconsistent grouping results between SCL-based assigning and BLAST-based physical mapping were excluded before mapping (S2 Table.). Finally, a genetic map (Fig. 1 and S1 Fig.), designated as the BY map, was constructed with a total of 224 simple PCR-based markers (including 129 InDels and 95 SSRs), the 14 remaining markers (11 InDels and 3 SSRs) were not integrated because of insufficient linkage. The BY map consisted of 13 linkage groups (LGs) covering a total genetic distance of 1249.77 cM with an average density of one framework marker for every 5.60 cM (Table 2 and S3 Table.). The number of mapped markers per LG ranged from 5 to 28 with an average of 17.23 markers. The largest and smallest genetic distance between two markers was 30.45 cM and 0.10 cM, respectively. This interspecific map that purely based on InDel and SSR would be useful for both of the basic and applied research for pepper in the future.

**Table 2:** Statistics of the pepper BY map based on InDel and SSR markers.

Linkage group	Chromosome	No. of markers				Marker distance (cM)			Map length	
		InDel	SSR	InDel and SSR	Distorted	Average	Min	Max	Genetic (cM)	Physical ^a^ (Mb)
LG1	P1	11	10	21	11	3.93	0.22	13.59	78.57	291.27
LG2	P2	17	11	28	4	3.54	0.10	13.73	95.52	140.55
LG3	P3	12	9	21	10	7.16	0.57	26.58	143.26	251.91
LG4	P4	9	6	15	5	7.24	0.68	21.95	101.34	204.67
LG5	P5	9	6	15	2	7.83	0.67	26.00	109.66	202.72
LG6	P6	10	7	17	3	7.58	0.97	17.37	121.29	216.29
LG7	P7	7	4	11	8	6.09	0.60	30.45	60.91	147.49
LG8	P7	1	4	5	3	4.19	1.20	8.25	16.74	7.15
LG9	P8	13	14	27	25	3.98	0.58	13.86	103.47	130.30
LG10	P9	8	7	15	1	7.89	1.09	17.93	110.48	228.33
LG11	P10	9	1	10	8	11.11	1.12	30.13	100.03	180.06
LG12	P11	7	6	13	6	7.16	0.75	17.53	85.92	185.61
LG13	P12	16	10	26	5	4.90	0.33	26.61	122.56	226.55
Total	-	129	95	224	91	5.60	-	-	1249.77	2412.90

^a^ Physical distance spanned by the BY map.

In this study, 91 out of 224 (40.63%) markers showed distorted segregation at an *P*<0.05 significance level (Table 2 and S3 Table.), which was considerably higher than that of several intraspecific populations [9,10,58] but similar to interspecific crossings [19,20]. Moreover, five segregation distortion regions (SDRs) were found on LG1, LG7, LG9 and LG11 (Fig. 1 and S3 Table.). Interestingly, all of the marker alleles within SDRs on LG7 (= P7) and LG11 (= P10) were skewed toward the female line BA3, whereas almost all were associated with the hybrid (F_1_) of the parental lines on LG1 (= P1) and LG9 (= P8). This indicates that there may be some segregation distorted factors in these regions [59]. For example, the incomplete chromosome pairing that resulted from reciprocal translocation between chromosome P1 and P8 [15] and the reduced recombination in this interspecific cross may be important determining factors in the selection of heterozygous genotypes in these areas.

### Comparison of genetic and physical maps

Since the 12 pseudo chromosomes (nominated as P1~ P12 [15]) of the Zunla-1 reference genome were built previously [1], the 13 LGs of the present BY map were successfully assigned to the 12 corresponding chromosomes in the haploid pepper genome based on 184 anchored markers including the 116 InDels and 68 SSRs (S1 and S2 Tables.). Even though the density of one marker per 14.96 Mb was low relative to the Zunla-1 reference genome (3.35 Gb), the total physical distance spanned by the BY map was 2,412.90 Mb (Table 2), which accounted for 72.03% of the Zunla-1 reference genome. Based on the comparative analysis (Fig. 1), P7 was found to be divided into two LGs (LG7 and LG8) due to insufficient linkage between them. Additionally, the high degree of consistency between the genetic and physical positions on P2, P8, P10 and P12 indicated these chromosomes are relatively conserved between the *C*. *annuum* and *C*. *frutescens* genomes [15]. Meanwhile, many inversions, especially for P1, could account for the variations between *C*. *frutescens* and *C*. *annuum*. Regardless, the relatively high whole genome coverage suggests that the BY map can serve as a basic reference map for targeted saturation and genetic applications in the future.

### Genetic analysis of Nle

**Fig 2:**
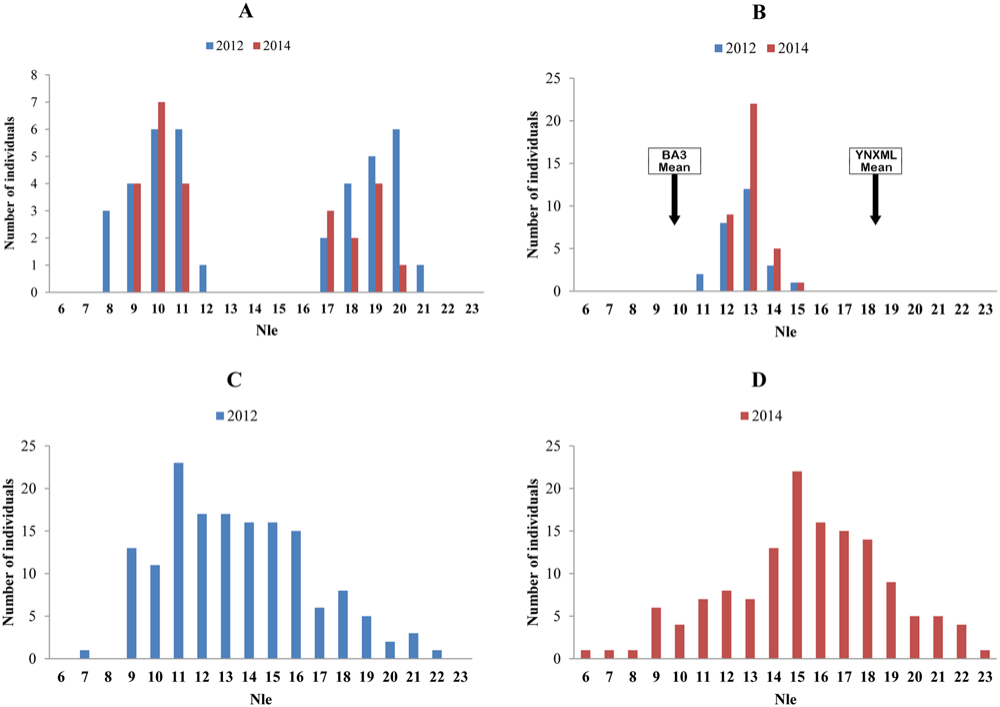
Frequency distribution of Nle for different populations in 2012 and 2014. A: Parental lines (Left: BA3, Right: YNXML), B: F_1_ population, the mean of parental lines were shown with black arrows, C and D: F_2_ populations in 2012 and 2014, respectively.

Nle is one of the component traits of the complex trait earliness, which is tightly correlated with flowering time [31,37]. There were significant differences (*P* < 0.01) in Nle between BA3 and YNXML (Table 3 and Fig. 2A), which led directly to a flowering time that occurred 10 ~ 15 days later in YNXML. The Nle that in the BA3 × YNXML hybrid (F_1_) is maternal biased (Fig. 2B), and it showed significantly continuous variation in the two subsets of F_2_ progenies with unimodal distribution (Figs. 2C and 2D). The heritability estimates of Nle were as high as 87.89% and 93.79% in 2012 and 2014, respectively (Table 3). These data indicate that Nle is suitable for artificial selection and is under polygenic control.

**Table 3:** Comparison of Nle in different generations and years.

Years	BA3			YNXML			BA3 × YNXML F1			BA3 × YNXML F2			Broad heritability (%)
	N	Mean	SD	N	Mean	SD	N	Mean	SD	N	Mean	SD	
2012	20	9.90	1.17	18	19.00	1.14	26	12.73	0.92	154	13.52	3.10	87.89
2014	15	10.00	0.76	10	18.30	1.06	37	12.95	0.70	139	15.40	3.43	93.79

### Identification of the QTLs affecting Nle

By combining the new BY genetic map with the phenotypic value (S4 Table.) of Nle from the subset of the F_2_ population (n = 154) in 2012, a total of 6 QTLs, including one major (named *Nle2*.*2*) and 5 minor QTLs (*Nle2*.*1*, *Nle7*.*1*, *Nle10*.*1 Nle10*.*2* and *Nle11*.*1*), were detected on P2, P7, P10 and P11 with both ICIM and CIM methods (Table 4). The results were consistent with the classic statistical genetic analysis in the present study as well as earlier studies [39]. Phenotypic variation explained by these QTLs varied between 2.09 and 51.63%. Except for the *Nle11*.*1* identified by the CIM, the Nle-increasing alleles within the remaining 5 QTLs were all from the parent YNXML (Table 4).

**Table 4:** QTLs for Nle identified by two mapping methods in the present study.

Year	Method	QTL^a^	Chromosome	Position	Interval^b^	LOD	PVE^c^ (%)	Add	Dom
2012	ICIM	*Nle2.1*	P2	10.00	Hpms1_106—CIDH197	3.53	5.48	0.42	-1.29
		*Nle2.2*	P2	79.00	EPMS677—CIDHjw1_24	22.79	48.32	3.16	-0.46
		*Nle7.1*	P7	42.00	CIDH66—Hpms1_166	3.17	4.55	0.78	-1.04
		*Nle10.1*	P10	36.00	CIDH607—CIDH619	2.90	5.19	1.02	-0.12
	CIM	*Nle2.1*	P2	10.20	CIDH197—PSE342	4.24	7.59	0.52	-1.36
		*Nle2.2*	P2	78.80	EPMS677—CIDHjw1–24	19.80	51.63	2.98	-0.84
		*Nle10.2*	P10	27.00	CIDH356—CIDH985	2.52	2.09	0.79	0.34
		*Nle11.1*	P11	57.60	CIDH799—CIDH146	2.54	3.32	-1.02	-0.21
2014	ICIM	*Nle2.2*	P2	82.00	CIDHjw1_24—CIDHjw2_2	26.53	58.79	3.78	1.21
	CIM	*Nle2.2*	P2	82.30	CIDHjw2_2—CIDHjw2_6	29.68	31.14	3.87	1.42

^a^ Two QTLs from the P10 were named *Nle10.1* and *Nle10.2*, respectively, because the genetic distance between them was over 5 cM.

^b^ The marker that was closer to the peak of LOD was unlined.

^c^ PVE, phenotypic variation explained by the QTL.

**Fig 3:**
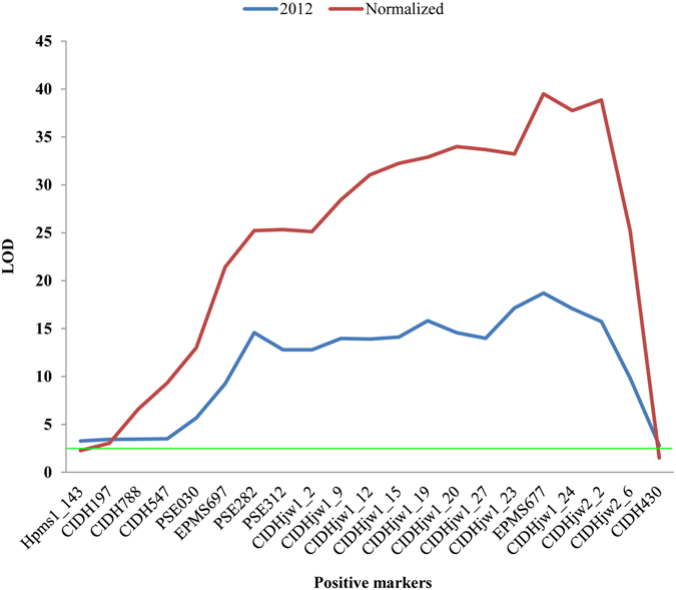
Likelihood profile comparison of single marker analysis with phenotypic data from 2012 and normalized, respectively. The threshold value of LOD = 2.5 was showed with green line.

To evaluate the QTL results, another subset of the F_2_ population (n = 147) was genotyped using a set of selective markers (n = 111) from the BY map and another map consisted of 13 LGs with 94 markers was constructed (unpublished data). After collecting the Nle phenotypic data from 139 out of 147 individuals planted in 2014, the major QTL, which was assumed to be equal to *Nle2*.*2* since the LOD peak markers EMPS677 and CIDHjw2_2 were tightly linked with physical distance of ~882.07 kb, was repeatedly identified using either ICIM or CIM method (Table 4). The results showed that the effect of *Nle2*.*2* on controlling the Nle was less affected by the environment and were consistent with the characterization of high heritability of this trait (Table 3). In addition, single marker analysis (SMA) was also carried out on a dataset that normalized from the phenotypic data of two years (2012 and 2014) with 21 selected positive markers (total number of the positive makers is 28) from the 2012 analyses. Plot of LOD (Fig. 3) showed that both peaks of 2012 and normalized were agreement with the results of ICIM and CIM indicating again that the environment has little influence on the Nle.

Previously, QTL analysis for Nle was conducted using different populations [31,40,44–46], most of which were intraspecific populations (Table 5). Among these studies, the cross used for QTL detection that was most similar to the current study was “B_9431_ × H108”, which is also an interspecific cross between *C*. *annuum* and *C*. *frutescens*. In addition, a total of 3 QTLs were detected on LG1 (= P6), LG7 (= P5) and LG22 (= P2) [46]. Interestingly, the QTL with the highest PVE (12.6%) was on P2 and one of its flanking markers, the CAMS-327, was found approximately 477.15 kb and 1,359.00 kb downstream of our two LOD peak markers (CIDHjw2_2 and EMPS677). However, the relationship between the two major QTLs requires further research. In summary, the QTLs affecting Nle were detected on all chromosomes except for P9 in *Capsicum* so far (Table 5), and there is at least one major QTL underlying the significantly natural variation in Nle between *C*. *annuum* and *C*. *frutescens* on chromosome P2.

**Table 5:** Comparison of mapped QTLs for Nle among studies.

Cross	Population type	Size	Number of QTLs	Chromosomes (LGs)	PVE(%)	References
BA3 × YNXML	Interspecific F2	154+139	6	P2, P7, P10, P11	2.1 ~ 58.8	This study
83–58 × perennial	Intraspecific RIL	122	3	P1, P2, P6	7.2 ~ 21.2	[45]
H3 × 83–60	Intraspecific RIL	100	8	P1, P2, P3, P4, P7, P8, P12	18.5 ~ 55.1	[45]
B_9431_× H108	Interspecific F2	180	3	LG1, LG7, LG22	6.4 ~ 12.6	[46]
YW × CM334	Intraspecific RIL	149	2	P3, P12	-	[40]
CW × LS2341	Intraspecific DH	94	2	P12, LG8	20.0 ~ 33.0	[31]
YW × CM334	Intraspecific RIL	297	4	P3, LG38 (= P11), LG45 (= P3), LG47	4.0 ~ 11.0	[44]

^a^ LG1, LG 7 and LG22 from the [46] were assigned onto the P6, P5 and P2 of the Zunla-1 genome by BLAST tool, respectively.

^b^ LG8 from the [31] were suggestively assigned onto P1 in spite of most of the marker were anchored on P0.

### Candidate genes in the *Nle2*.*2* region

Through integrating the QTL results from the two years (Table 4), we can delimit a loose candidate region for the major QTL *Nle2*.*2* into an interval between the marker EPMS677 and CIDHjw2_6 with physical distance of ~2.76 Mb on chromosome P2. A total of 153 protein coding genes (S5 Table.), including 37 new genes without homologs in public database, were predicted to embed in this region based on the current annotations of the Zunla-1 reference genome (http://peppersequence.genomics.cn). Even though two of the six flowering related genes reported previously in pepper, *CaS* (KC414761, equal to *Capana02g001854*) and *Ca-AN* (FJ190669, equal to *Capana02g002328*), were both mapped/anchored on the chromosome P2 as well [33,34], they are not included in the *Nle2*.*2* candidate region. On the other hand, because 12 out of the 153 genes that were found to be homologous to the flower/inflorescence development related proteins of Arabidopsis (Table 6), and consequently they were recommended as important candidate genes for the major QTL *Nle2*.*2* of pepper. Especially for *Capana02g003062*, which is homologous to the Arabidopsis *AP2* gene, as well as *Capana02g003067* and *Capana02g003070*, both of which are the homologs of Arabidopsis *CLF*. More importantly, both of *AP2* and *CLF* were important members of the flowering time pathway in Arabidopsis. For example, earlier studies reported that *AP2* was involved in the specification of floral organ identity, establishment of floral meristem identity and suppression of floral meristem indeterminacy [60–62]. In addition, *CLF* was also found to have effects on the vegetative phase to reproductive phase transition of meristem [63]. Taken these together, we suppose that *Nle2*.*2* is possibly a new member that participated in the flowering time regulation pathway of pepper and mainly controls the natural variation with respect to Nle in *Capsicum* population. The findings in present study would not only be useful for the isolation of genes controlling the initiation of flower primordia, but also provided insights into the molecular regulation of flowering time in pepper.

**Table 6:** List of 12 important candidate genes for the *Nle2.2* of pepper and homologs of Arabidopsis.

Gene ID	Position on chromosome P2		Homologous gene symbol	Descriptions
	Start	Stop		
*Capana02g003062*	154,819,441	154,822,016	*AP2*	Floral homeotic protein APETALA 2
*Capana02g003067*	154,918,269	154,941,151	*CLF*	Histone-lysine N-methyltransferase CLF
*Capana02g003070*	154,981,807	155,004,727	*CLF*	Histone-lysine N-methyltransferase CLF
*Capana02g003079*	155,444,041	155,447,464	*SPT*	Transcription factor SPATULA
*Capana02g003089*	155,587,450	155,588,112	*HEC2*	Transcription factor HEC2
*Capana02g003131*	156,190,290	156,194,955	*ANP1*	Mitogen-activated protein kinase kinase kinase ANP1
*Capana02g003133*	156,215,286	156,216,230	*LBD36*	LOB domain-containing protein 36
*Capana02g003199*	157,107,846	157,110,095	*COL2*	Zinc finger protein CONSTANS-LIKE 2
*Capana02g003200*	157,118,313	157,120,062	*COL2*	Zinc finger protein CONSTANS-LIKE 2
*Capana02g003201*	157,124,787	157,126,407	*COL2*	Zinc finger protein CONSTANS-LIKE 2
*Capana02g003223*	157,449,873	157,469,179	*BIG*	Auxin transport protein BIG
*Capana02g003224*	157,469,217	157,471,764	*BIG*	Auxin transport protein BIG

## Conclusions

An interspecific genetic map of pepper, comprising of a total of 224 purely anchored markers including InDel and SSR, was constructed. Assignment of the LGs to corresponding chromosomes indicated the relatively high coverage and confirmed the variations of *Capsicum* genomes. One major QTL (*Nle2*.*2*) influencing flowering time was identified on the chromosome P2 in 2012 and confirmed in 2014. Based on the annotations of Zunla-1 reference genome, 153 protein coding candidate genes were suggested. Hence, the map, QTLs and candidate genes obtained by the present study will be useful for future basic and applied research with respect to flowering time in *Capsicum*.

## Supporting Information

S1 Fig Graphical genotypes of the 154 F_2_ individuals with the 224 markers mapped on the BY map. A (red): homozygote as BA3, H (yellow): heterozygote as F1, B (dark blue): homozygote as YNXML, C (light blue): not A, D (brown): not B, U (gray), missing.
(TIF)

S1 Table The information on the polymorphic Indel markers genotyped in the present study. (XLSX)

S2 Table The primer sequences and BLAST-based chromosome mapping information of SSR markers from different sources. (XLSX)

S3 Table The genetic position and segregation distortion of 224 markers mapped on the BY map. (XLSX)

S4 Table Raw data of *Nle* for different pepper populations in 2012 and 2014. (XLSX)

S5 Table List of 153 candidate genes located in the QTL *Nle2*.*2*. (XLSX)
