# Supplementary figures and images for: Construction of an Interspecific Genetic Map Based on InDel and SSR for Mapping the QTLs Affecting the Initiation of Flower Primordia in Pepper (Capsicum spp.)
Source: PLoS One. 2015 Mar 17;10(3):e0119389. doi: 10.1371/journal.pone.0119389 (PMC4363154; doi:10.1371/journal.pone.0119389)

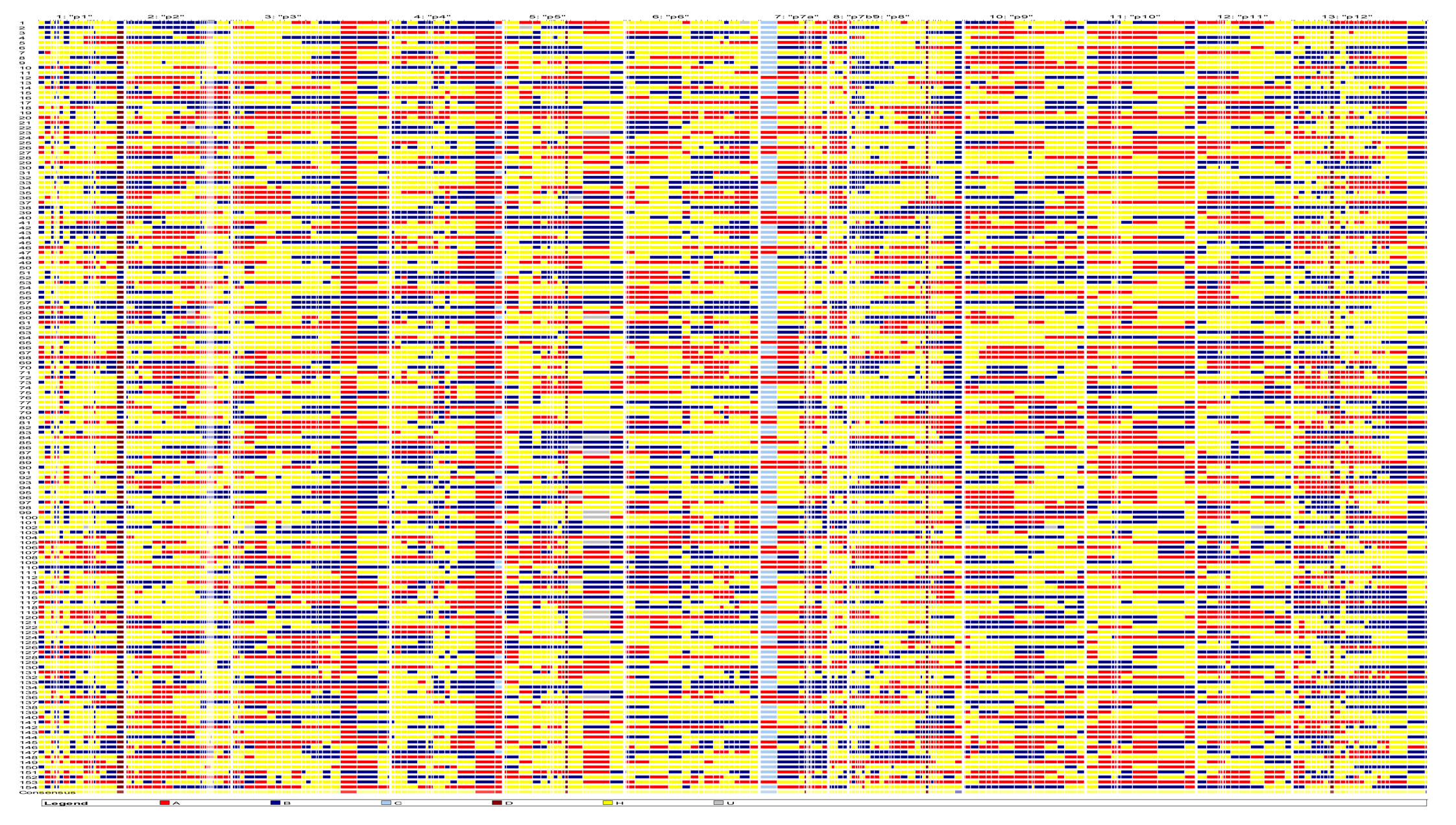

Supplement: S1 Fig — A (red): homozygote as BA3, H (yellow): heterozygote as F1, B (dark blue): homozygote as YNXML, C (light blue): not A, D (brown): not B, U (gray), missing. (TIF) [file pone.0119389.s001.tif]
